# Supplementary material for: Unveiling the Molecular Driving Forces of Pollutant Extraction by Hydrophobic Eutectic Solvents
Source: J Phys Chem B. 2026 Sep 29;130(40):10571–81. doi: 10.1021/acs.jpcb.6c04088 (PMC13656732; doi:10.1021/acs.jpcb.6c04088)
Supplement: Supplementary file 1 [file jp6c04088_si_001.pdf]

Supplementary Information: Unveiling the Molecular Driving Forces of Pollutant Extraction by Hydrophobic Eutectic Solvents

Sara Gómez,[*,†] Usman Ali,[‡] Alessia Muroni,[‡] Andrea Mele,[¶] Maria Enrica Di Pietro,[¶] and Tommaso Giovannini[*,‡]

†*Universidad Nacional de Colombia, Departamento de Química, Av. Cra 30 45-03, 111321, Bogotá, Colombia*

‡*Department of Physics, University of Rome Tor Vergata, Via della Ricerca Scientifica 1, 00133, Rome, Italy*

¶*Department of Chemistry, Materials and Chemical Engineering "Giulio Natta", Politecnico di Milano, Milano, 20133, Italy*

E-mail: sagomezam@unal.edu.co; tommaso.giovannini@uniroma2.it

S1 Monophasic MD simulation of BPA in water and in HES

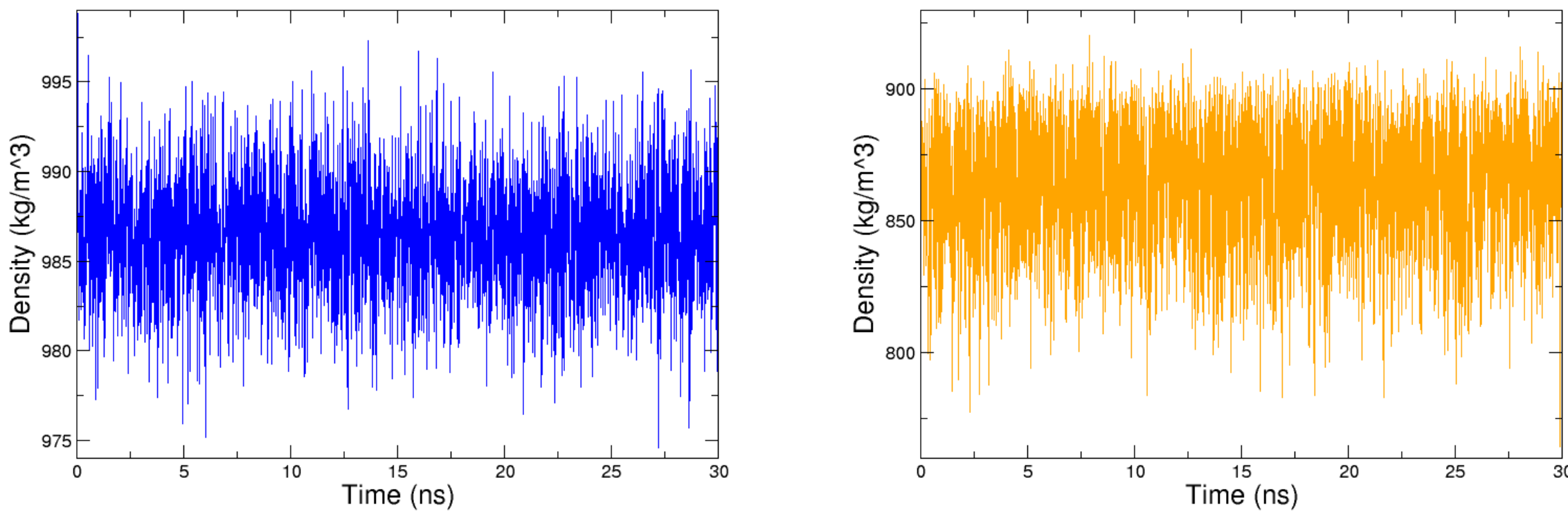

Figure S1: Total densities along the monophasic molecular dynamic simulation of BPA in water (left) and in TOPO:menthol HES (right).

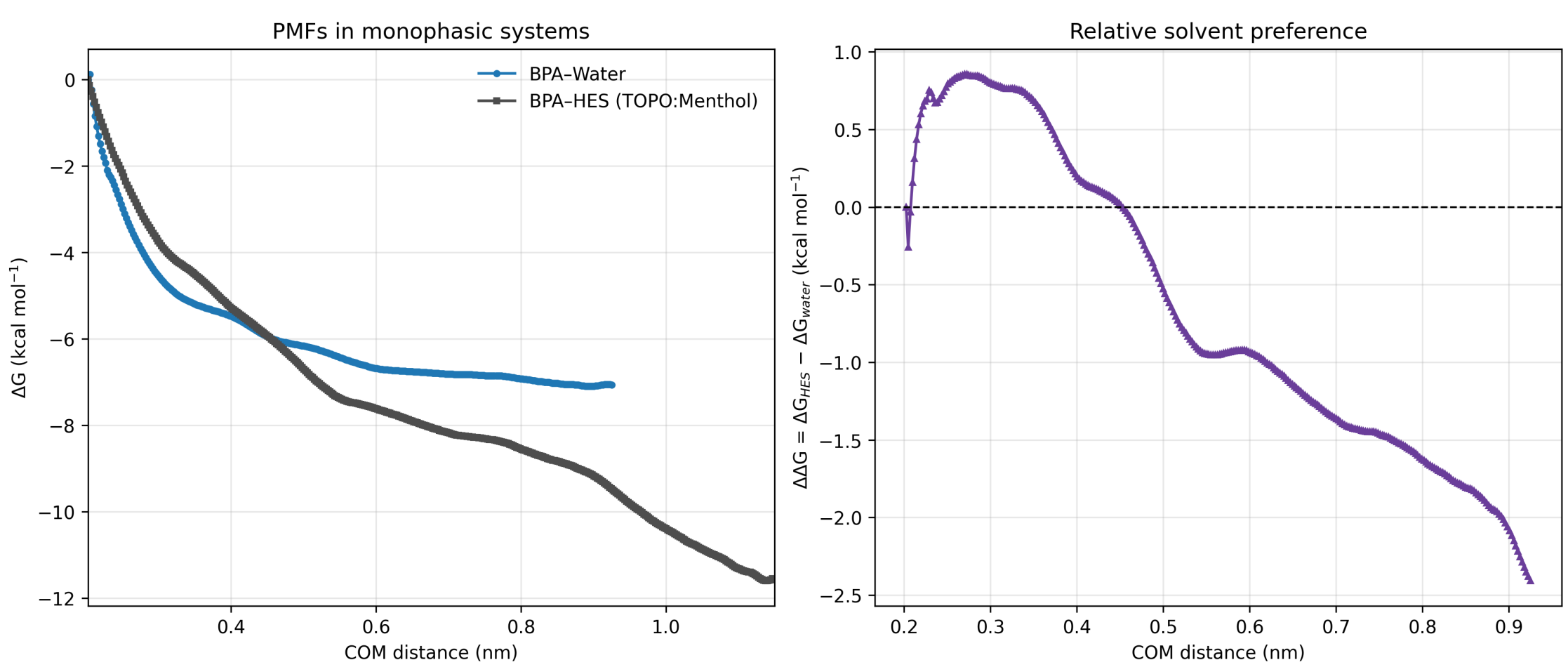

Figure S2: Potential of mean force (PMF) profiles and relative solvent preference for bisphenol A (BPA) in monophasic environments. (left) One-dimensional PMFs as a function of the center-of-mass (COM) distance between BPA and the reference solvent site, obtained from umbrella sampling and WHAM analysis, for BPA in water (blue) and in the hydrophobic eutectic solvent (HES, TOPO:menthol 1:2; dark gray). The HES PMF is shown up to 1.15 nm, corresponding to the full sampling range of the HES system, while the water PMF is limited to its accessible distance range. (right) Relative free-energy profile, defined as $\Delta G_{transfer} = \Delta G^{HES}_{binding} - \Delta G^{water}_{binding}$, evaluated only over the distance interval common to both systems. Negative values indicate preferential stabilization of BPA in the HES environment relative to water.

Table S1: Reduced clusters obtained from the BPA-in-water MD trajectory. For each cluster, the number of water molecules (# waters) included in the reduced model and its population along the trajectory are reported.

Cluster	# waters	Population (%)
BPA-in-Water$_1$	7	31.02%
BPA-in-Water$_2$	5	29.49%
BPA-in-Water$_3$	7	21.13%
BPA-in-Water$_4$	10	15.03%
BPA-in-Water$_5$	8	2.30%
BPA-in-Water$_6$	8	0.77%
BPA-in-Water$_7$	4	0.27%

Table S2: Reduced clusters obtained from the BPA-in-HES MD trajectory. For each cluster, the number of TOPO (# TOPO), MEN (# MEN), and HES molecules (# HES molecules)included in the reduced model, together with its population along the trajectory, are reported.

Cluster	# TOPO	# MEN	# HES molecules	Population (%)
BPA-in-HES$_1$	1	2	3	53.92%
BPA-in-HES$_2$	2	2	4	37.22%
BPA-in-HES$_3$	1	0	1	8.06%
BPA-in-HES$_4$	1	2	3	0.77%
BPA-in-HES$_5$	1	1	2	0.03%

Table S3: Reduced clusters obtained from the BPA-in-HES MD trajectory. For each cluster, the number of TOPO (# TOPO), dodecanoic acid (# DOD), and HES molecules (# HES molecules)included in the reduced model, together with its population along the trajectory, are reported.

Cluster	# TOPO	# DOD	# HES molecules	Population (%)
BPA-in-HES$_1$	1	1	2	60.28%
BPA-in-HES$_2$	1	1	2	32.92%
BPA-in-HES$_3$	1	1	2	6.80%

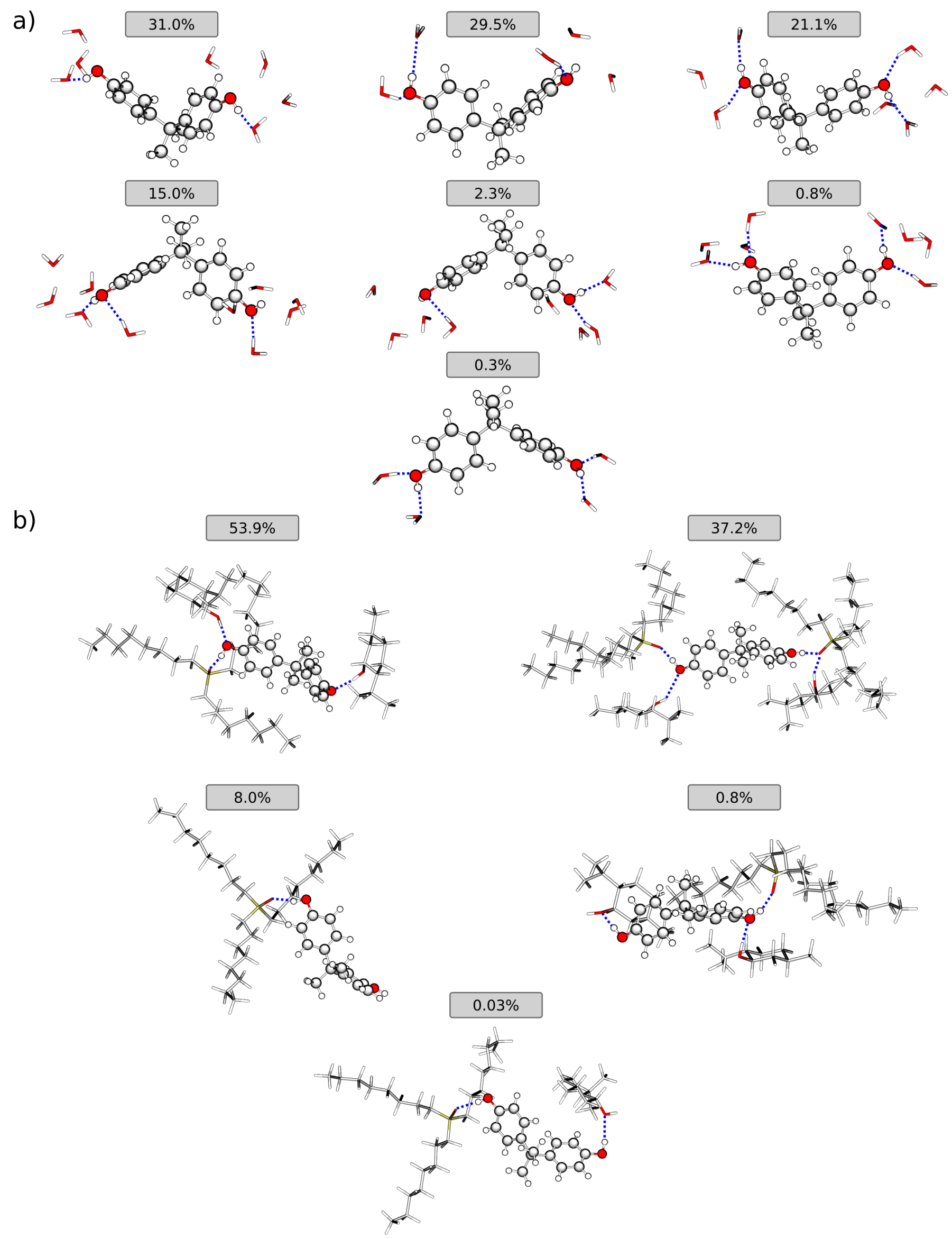

Figure S3: Graphical depiction of the reduced clusters extracted from BPA-in-Water (a) and BPA-in-HES (TOPO:menthol) (b) trajectories. Hydrogen Bonds are shown as blue dashed lines.

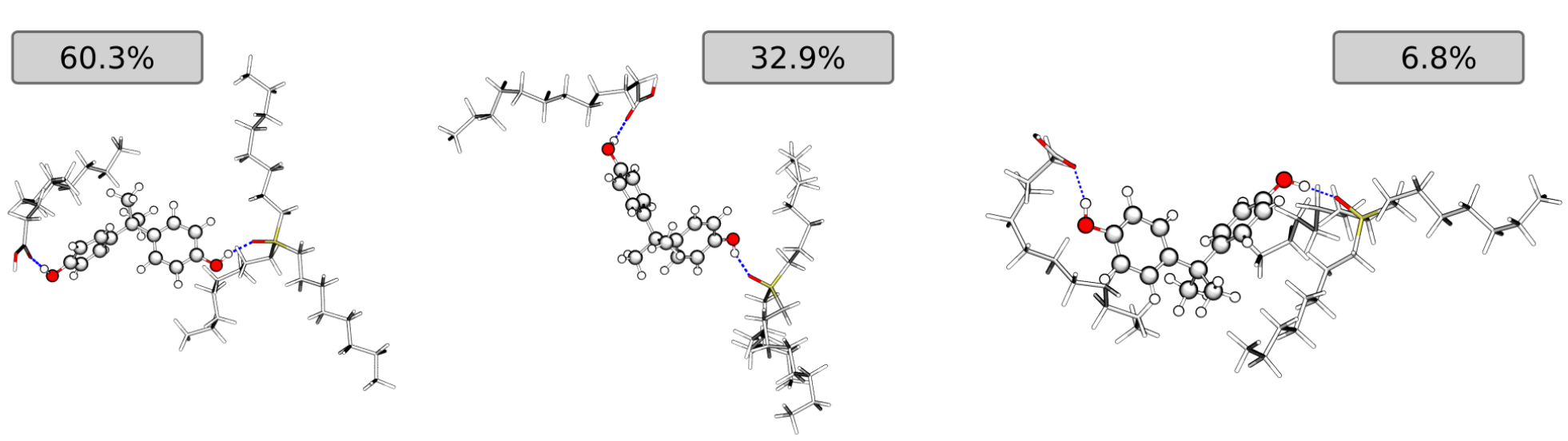

Figure S4: Graphical depiction of the reduced clusters extracted from BPA-in-HES (TOPO-dodecanoic acid) (b) trajectories. Hydrogen Bonds are shown as blue dashed lines.

S1.1 KS-FEDA Raw Data

Table S4: KS-FEDA for the clusters extracted from the MD simulations of BPA in pure water. The weighted energy contributions are obtained by a weighted average using the populations reported in Tab. S1.

Structure	E^{ele}	E^{ex-rep}	E^{pol}	E^{disp}	E^{int}
BPA-in-Water$_1$	-28.352	34.139	-9.051	-12.900	-16.165
BPA-in-Water$_2$	-32.006	43.147	-8.673	-13.499	-11.031
BPA-in-Water$_3$	-34.273	38.311	-10.330	-14.988	-21.278
BPA-in-Water$_4$	-40.342	47.732	-11.912	-21.619	-26.141
BPA-in-Water$_5$	-46.137	64.144	-16.979	-20.048	-19.021
BPA-in-Water$_6$	-39.255	45.681	-12.445	-16.803	-22.822
BPA-in-Water$_7$	-34.052	37.920	-10.584	-11.985	-18.701
weighted	-32.991	40.509	-9.852	-15.020	-17.354

Table S5: KS-FEDA for the clusters extracted from the MD simulations of BPA in TOPO:menthol HES. The weighted energy contributions are obtained by weighted average using the populations reported in Tab. S2.

Structure	E^{ele}	E^{ex-rep}	E^{pol}	E^{disp}	E^{int}
BPA-in-HES$_1$	-35.554	47.137	-12.902	-28.820	-30.138
BPA-in-HES$_2$	-53.165	77.695	-25.635	-31.140	-32.245
BPA-in-HES$_3$	-18.548	22.146	-7.787	-11.055	-15.244
BPA-in-HES$_4$	-37.804	48.393	-15.360	-23.365	-28.135
BPA-in-HES$_5$	-21.503	24.145	-7.294	-17.051	-21.704
weighted	-40.751	56.499	-17.246	-28.206	-29.704

Table S6: KS-FEDA for the clusters extracted from the MD simulations of BPA in TOPO:dodecanoic acid HES (see also Fig. S4). The weighted energy contributions are obtained by weighted average using the populations reported in Tab. S3.

Structure	E^{ele}	E^{ex-rep}	E^{pol}	E^{disp}	E^{int}
BPA-in-HES$_1$	-38.355	54.904	-17.075	-22.111	-22.637
BPA-in-HES$_2$	-31.472	38.442	-13.006	-14.203	-20.239
BPA-in-HES$_3$	-38.604	55.226	-16.926	-27.294	-27.598
weighted	-36.106	49.507	-15.725	-19.860	-22.185

S2 Biphasic MD analyses

S2.1 BPA in a mixture of water-HES TOPO:menthol

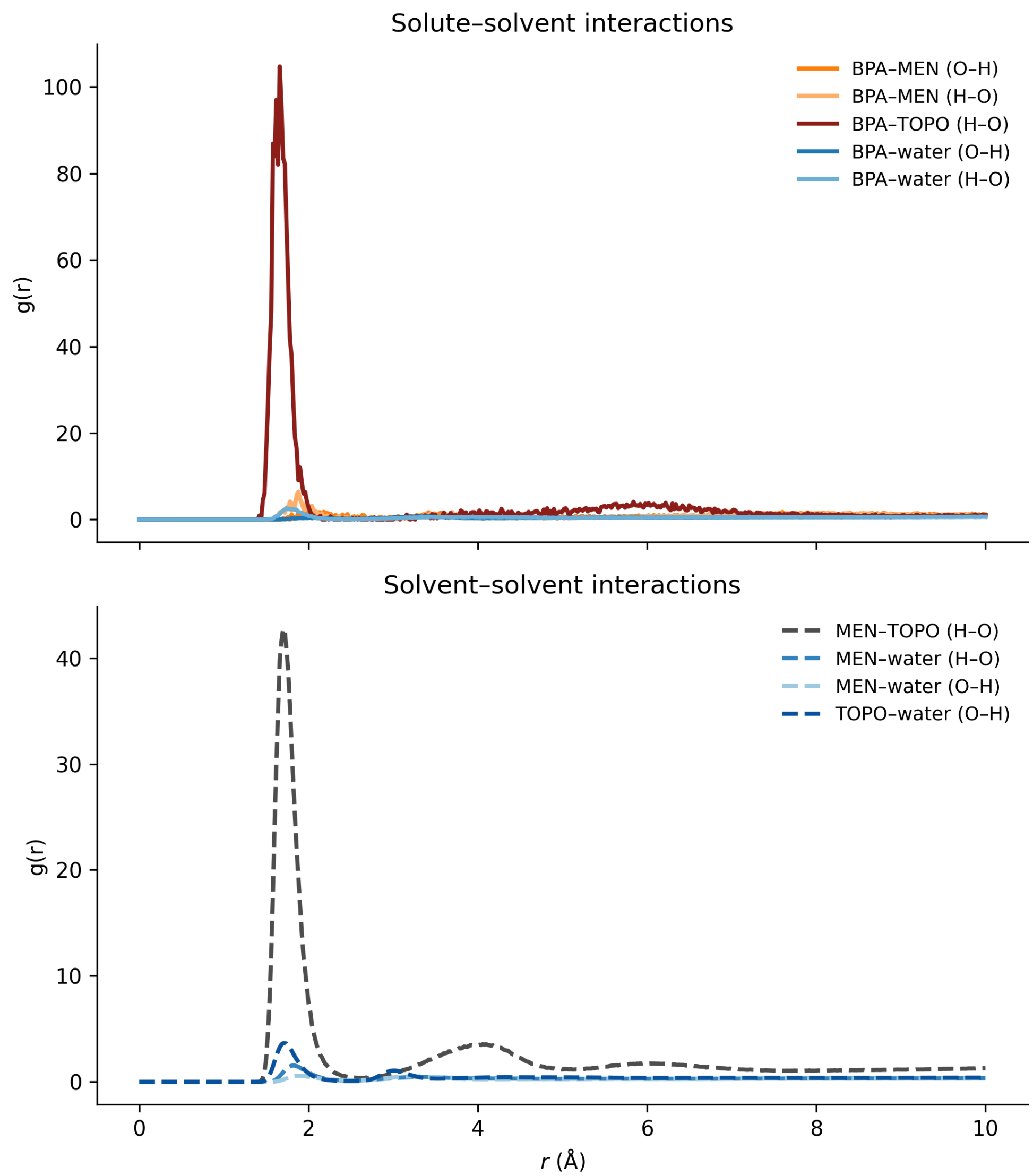

Figure S5: Radial distribution functions (RDFs) for the biphasic water–HES system containing BPA. (**Top**) Solute–solvent RDFs describing hydrogen-bond–relevant atom pairs between BPA and the surrounding solvents, including BPA–menthol, BPA–TOPO, and BPA–water interactions. (**Bottom**) Solvent–solvent RDFs for interactions among HES components and between HES and water. Distinct short-range structuring is observed for BPA–HES interactions compared to BPA–water, consistent with the preferential solvation of BPA within the HES-rich phase in the biphasic system.

S2.2 BPA in a mixture of water-HES TOPO:dodecanoic acid

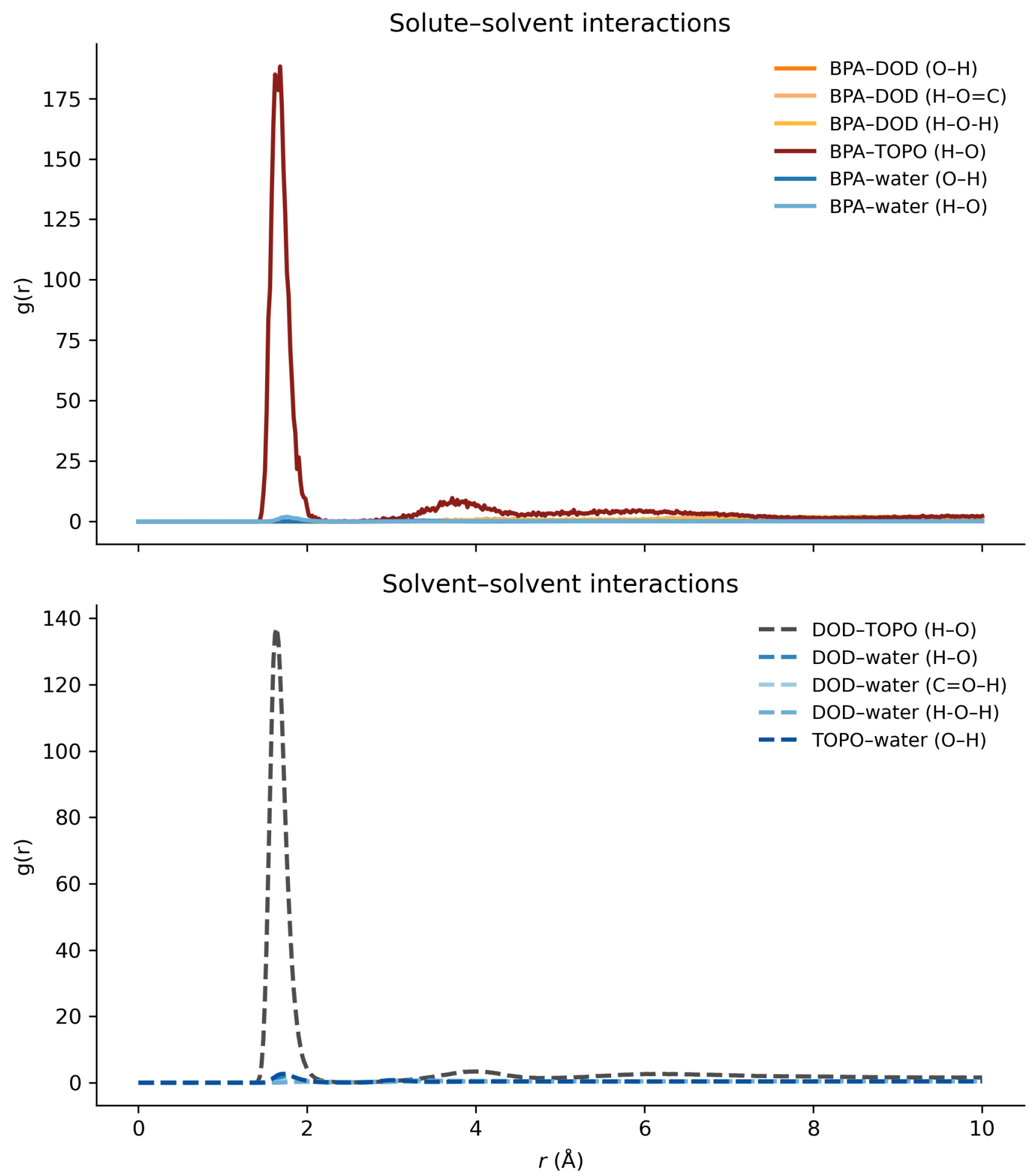

Figure S6: Radial distribution functions (RDFs) extracted from the MD production stage of the biphasic water + TOPO:dodecanoic acid (1:1) HES system containing BPA. (**Top**) Solute–solvent RDFs describing hydrogen-bond–relevant atom pairs between BPA and the surrounding solvents, including BPA–dodA, BPA–TOPO, and BPA–water interactions. (**Bottom**) Solvent–solvent RDFs for interactions among HES components and between HES and water. Distinct short-range structuring is observed for BPA–HES interactions compared to BPA–water, consistent with the preferential solvation of BPA within the HES-rich phase in the biphasic system.

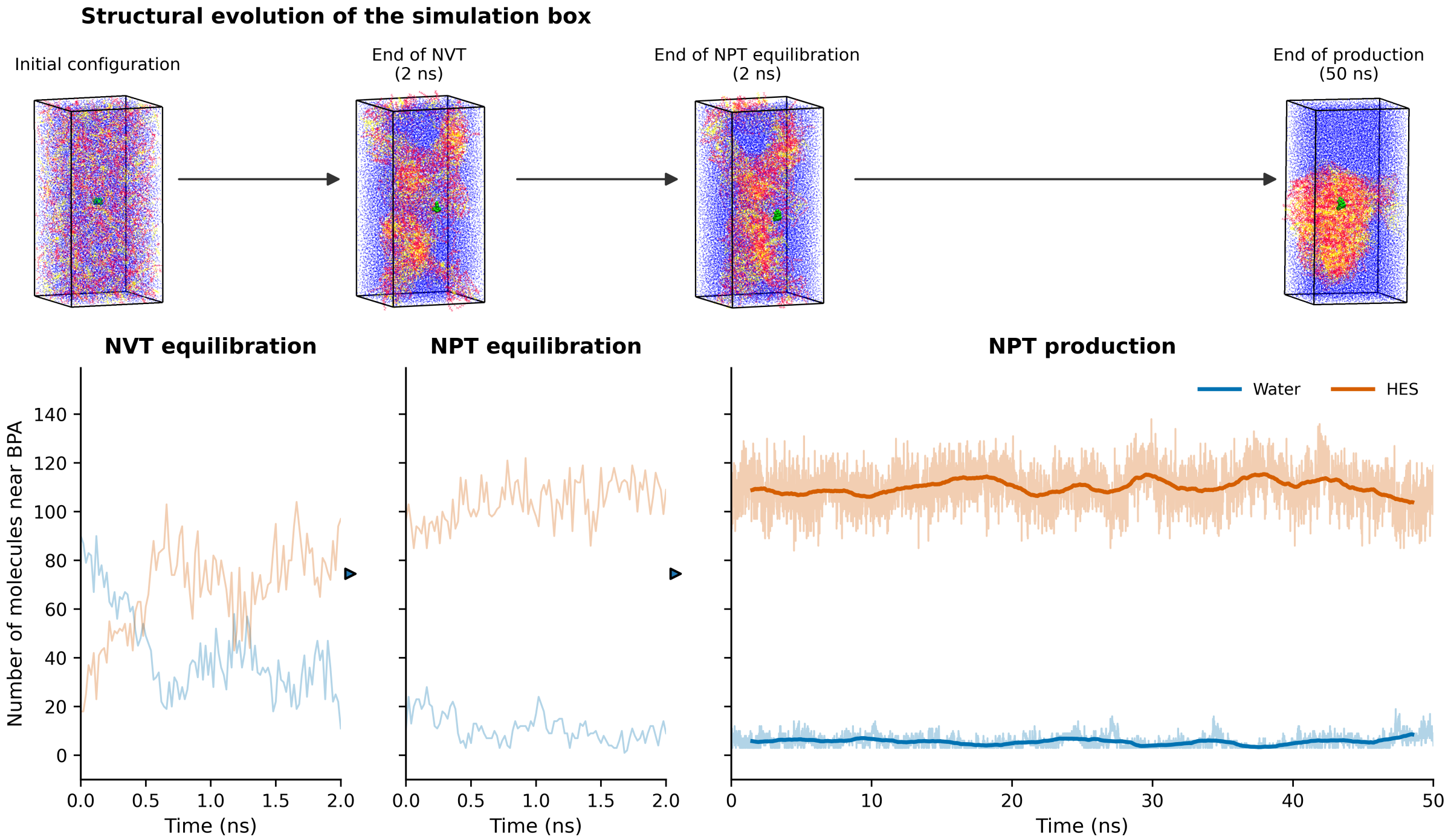

Figure S7: Evolution of the BPA solvation environment during the NVT and NPT equilibration stages and the subsequent 50 ns NPT production simulation in the water + TOPO:dodecanoic acid (1:1) HES system. The number of water molecules (blue) and total HES molecules, including both TOPO and dodecanoic acid (orange), within the selected contact distance of BPA is shown as a function of simulation time for each stage. The raw trajectories are shown with reduced opacity, whereas the solid lines represent moving averages. Representative simulation-box configurations are shown above the corresponding stages to illustrate the structural reorganization of the system.
